# Agenesis of all permanent maxillary incisors: A rare clinical case with an interdisciplinary solution

**DOI:** 10.4317/jced.54698

**Published:** 2018-04-01

**Authors:** Fernando-Cesar Torres, Claudio-Fróes de Freitas, Diego-Vianez Pereira, Tarcila Triviño, Acácio Fuziy, Fernando-Akio Maeda

**Affiliations:** 1 DDS, MSc, PhD. Associate Professor Doctor of the Master’s program in Odontology, University of São Paulo City (UNICID), São Paulo, Brazil; 2 DDS, MSc, PhD. Head Professor of the Master’s program in Odontology, University of São Paulo City (UNICID), Brazil; 3 DDS, MSc. Private practice, São Paulo, Brazil

## Abstract

**Background:**

Clinical cases involving agenesis of all four maxillary incisors are rare, with no previous reports in the literature.

**Case report:**

The present case report describes an orthodontic treatment combined with esthetic dentistry in a 10-year-old girl with agenesis of all four permanent maxillary incisors, anterior crossbite, permanence of deciduous maxillary canines and transmigration of permanent maxillary canines into the region of the maxillary central incisors. For this case, it was decided on space closure using a fixed orthodontic apparatus and reshaping of the first premolars transforming them into canines. Porcelain veneers were used on the permanent and deciduous canines, substituting the maxillary central and lateral incisors, respectively. Regarding outcome, there was an improvement in facial profile, correction of the anterior crossbite, satisfactory intercuspidation of the teeth and significant esthetic improvement in smile. Maintaining the patient´s natural dentition also kept the bone plate intact for future placement of implants to substitute maxillary deciduous canines at the appropriate age.

**Conclusions:**

Interdisciplinary planning combining orthodontics and esthetic dentistry was key in resolving this case.

** Key words:**Tooth agenesis, upper incisors, orthodontic treatment.

## Introduction

Dental agenesis or hypodontia is one of the most common developmental anomalies of human dentition. Hypodontia is defined as the absence of between one and five teeth, excluding the third molars (1). Its etiology remains unclear, although most cases involve genetic autosomal dominant inheritance. Studies comparing monozygotic twins suggest the influence of environmental factors (2,3). Trauma, infections (rubella, osteomyelitis), drugs, radiotherapy or chemotherapy can affect the growth of embryonic tooth cells.

Large disparities in the prevalence of dental agenesis have been reported in the literature ranging from 0.3 to 36.5% (4,5). The prevalence of agenesis of the maxillary lateral incisors ranks second only to the third molars and mandibular second premolar, and is 1.37 times higher in woman than in men (6). According to the meta-analysis of Polder *et al.* (6), the prevalence of agenesis of the maxillary lateral incisors is approximately 1.8% while of the maxillary central incisors is around 0.01% and considered rare by the authors.

Some clinical cases of a single missing maxillary central incisor, referred to as “solitary median maxillary central incisor”, have been reported in the literature (7-9). However, there are no reports in the literature of clinical cases of patients with agenesis of two maxillary central incisors, while cases with four missing maxillary incisors are rarer still.

In this study, we report a clinical case of a patient with agenesis of all four permanent maxillary incisors. The patient´s teeth were aligned, levelled, orthodontically repositioned and prosthetically rehabilitated without the use of implants, leading to a marked improvement in facial profile and smile esthetic.

## Case Report

- Diagnosis and etiology

**Figure 1:**
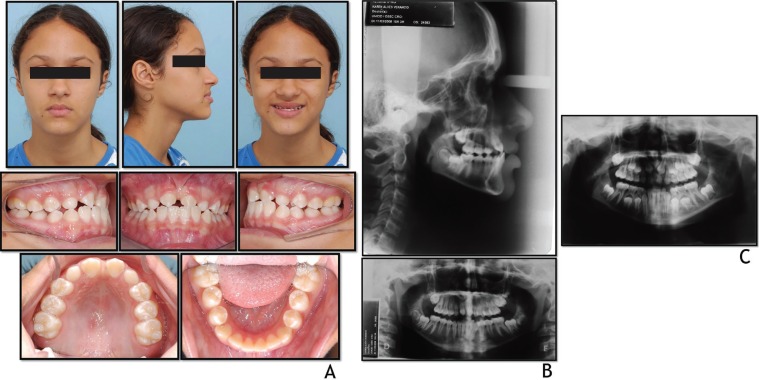
Pretreatment: A) facial and intraoral photographs; B) cephalometric and panoramic radiographs; C) Panoramic radiograph taken at 8 years: agenesis of the deciduous lateral incisors upper, permanent central and lateral maxilary incisors, and mesial ectopic eruption of the permanent maxillary canines.

A girl aged 10 years 4 months presented with a slightly concave profile, lack of upper labial support, highly accentuated peribuccal sulcus, good lip seal and acceptable facial asymmetry. The patient had a good overall state of health with no history of systemic disease, trauma or deleterious habits. The intraoral exam revealed: ½ Class II molar relationship, four missing permanent maxillary incisors, anterior crossbite, permanence of deciduous maxillary canines and transmigration of permanent maxillary canines into the region of the maxillary incisors (Fig. 1 A,B).

The patient sought treatment for poor anterior teeth esthetics and anterior cross-bite rendering the patient too embarrassed to smile. Analysis of the initial panoramic radiograph taken at 8 years 6 months disclosed the presence of 2 deciduous maxillary central incisors but absence of maxillary permanent incisor buds and transmigration of the maxillary canine buds mesially (Fig. 1C). The patient´s mother confirmed that the maxillary deciduous lateral incisors had always been missing. Panoramic radiographs and lateral teleradiography taken prior to orthodontic treatment disclosed almost complete eruption of the canines in the position of the maxillary central incisors and a poor relationship of the arches owing to missing anterior teeth, i.e., due to short maxillary arch, with no significant sagittal maxillomandibular discrepancy.

- Treatment objectives

The following treatment objectives were established: (1) to establish an esthetic facial profile; (2) to resolve the anterior crossbite; (3) to obtain a stable occlusal relationship; and (4) to improve dental esthetics, establishing an esthetic smile.

- Treatment alternatives

Three treatment options were presented to the parents. First, distalization of the maxillary molars using an extrabuccal arch to correct the molar relationship, extraction of the deciduous canines, moving of the permanent canines into the position of the deciduous canines, fabrication of temporary prostheses until the patient reaches sufficient bone maturity to allow placement of four implants and fabrication of prostheses in the region of the four maxillary incisors. This option was rejected by the parents for financial reasons and owing to the lengthy orthodontic treatment required until implant placement.

The second option involved mesialization of the maxillary molars to achieve a Class II molar relationship, whereby the first two maxillary premolars substitute the maxillary canines, the maxillary canines substitute the maxillary central incisors, with the required esthetic and functional rehabilitation and extraction of the deciduous canines, and space opening for provisional teeth in maxillary lateral incisors until the patient reaches sufficient bone maturity to allow placement of two implants and fabrication of prostheses in the region of the two lateral incisors. This option was also rejected, mainly owing to the orthodontic treatment time required until placement of prostheses on implants.

The third option involved mesialization of the maxillary molars to achieve a Class II molar relationship, whereby the first two maxillary premolars substitute the maxillary canines, the maxillary canines substitute the maxillary central incisors and the deciduous canines are moved to the region of the lateral incisors, with all these teeth submitted to the required esthetic and functional rehabilitation. This third option was elected.

- Treatment progress

Treatment commenced by bracket bonding with the MBT prescription, banding of the first molars, taking care not to bond any brackets to deciduous canines thereby avoiding the risk of overload and exfoliation. The MBT prescription was chosen for its incisor bracket angulation, which can help correct the anterior crossbite. Maxillary central incisor brackets were bonded to maxillary canines, taking into account end of treatment, to allow better positioning of these teeth for torque, angulation and height.

**Figure 2:**
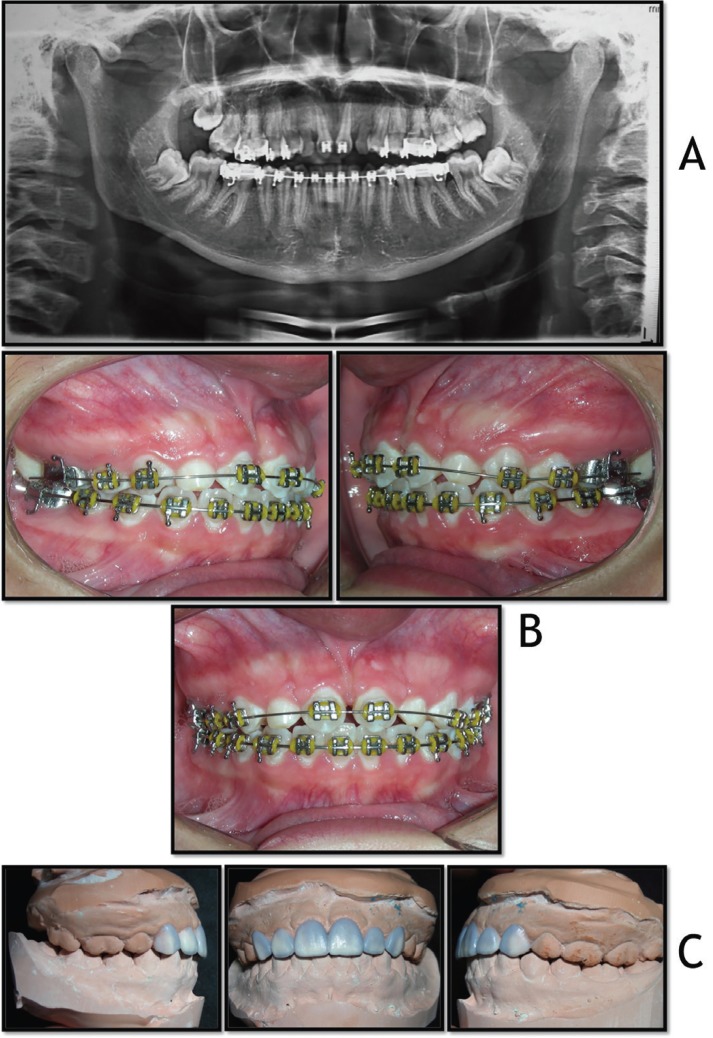
Progress: A) panoramic radiograph; B) intraoral photographs: the teeth were aligned and leveled, taking care not to bond any brackets to deciduous canines thereby avoiding the risk of overload and exfoliation. C) A mock-up performed.

In the first phases of the treatment, the teeth were aligned and leveled using 0.012-in and 0.016-in nickel-titanium wires (Fig. 2A). Nickel-titanium open-coil springs were employed to obtain the ideal spacing equivalent to the four maxillary incisors for future rehabilitation of the teeth. After round nickel-titanium wires, 0.016 X 0.020-in stainless steel wire were used, interspersed with rectangular section 0.017 x 0.025-in nickel-titanium wire, finishing with 0.019 x 0.025-in stainless steel wire. During this phase, the patient was instructed to use Class III intermaxillary elastics.

The whole fixed apparatus was then removed and during the same session, a 3 x 3 retainer was fabricated in the lower arch anchored by the lingual aspect of the anterior teeth. A mock-up was first performed (Fig. 2B), and after approval of the results, teeth 13 and 23, previously moved to substitute the maxillary central incisors, were prepared and fitted with feldspathic porcelain laminate veneers. Teeth 53 and 63 were also fitted with feldspathic porcelain veneers, assuming the position and function of the maxillary lateral incisors. Teeth 14 and 24 were reshaped with direct composite resins and assumed the function and position of the maxillary canines, with adjustment for group function when performing laterality.

- Treatment results

**Figure 3:**
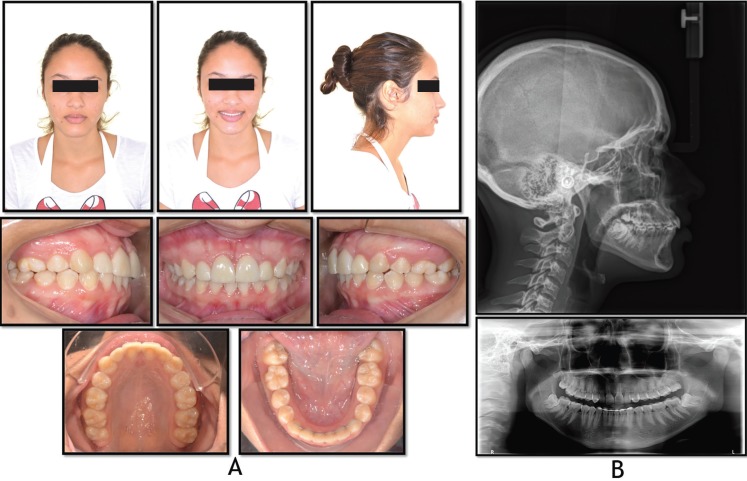
Posttreatment: A) facial and intraoral photographs; B) cephalometric and panoramic radiographs.

At end of treatment, all goals were achieved. There was an improvement in the patient´s facial profile from slightly concave to straight. The crossbite problem was resolved. Final intercuspidation of the teeth was highly satisfactory. The esthetic rehabilitation of all maxillary teeth restored the patient´s self-esteem owing to the major esthetic improvement (Fig. 3). The patient was asked whether the mandibular third molars could be extracted, since these were impacted and inclined mesially.

The patient was highly satisfied with the results obtained, particularly the esthetic and functional correction. However, the patient and her parents were advised about possible mobility of the deciduous canines and made aware of the likely need to replace these two teeth with implants at a later date.

## Discussion

Dental agenesis has a major functional and esthetic impact, exacerbated by the number of teeth involved. The consequences of agenesis can include the emergence of diastemas, infraocclusion of deciduous teeth, inadequate angulations of adjacent teeth, occlusal trauma, among others. The planning of these cases is invariably interdisciplinary, where the main doubt in treating dental agenesis cases typically hinges on the dilemma of space closure or space opening for prosthetic rehabilitation (10).

The closure or opening of spaces in agenesis cases depends on the space discrepancy in the arch, type of facial profile, presence of maxillary dental protrusion or retrusion, type of malocclusion, agenesis symmetry and size and shape of teeth to be moved. Another challenge is the need to carry out treatment in patients that are still growing, where early diagnosis at around 7-9 years favors treatment outcome because teeth can be moved so as to minimize potential functional and esthetic compromise, conferring better quality of life for the child (11,12).

The current prosthetic solution of choice for agenesis cases, and considered a more conservative approach, is implant-supported prosthesis (13), although this treatment is not always possible. Factors such as age, bone quantity and quality as well as space availability can preclude placement of this type of implant. There is a consensus that, in young patients, implants and their prosthesis should only be fabricated after growth is fully complete (14-16). Some studies show that, even after complete dental and skeletal development, infraocclusion and progressive mal-alignment in crowns can occur in the anterior region of the maxilla (14,16). One viable strategy is placement of implants as late as possible, conserving the bone structure by keeping the deciduous teeth, which often remain for longer periods in the absence of a permanent successor (17). For cases in which implants are unfavorable, fabrication of a fixed prosthesis may be a good solution.

In the clinical case reported in this study, the patient was of growing age and the maxillary canines exhibited a mesial eruption path, located close to the midline. The deciduous canines had medium root length but no mobility. Therefore, the option of replacing the missing teeth (11, 12, 21 and 22) with implants was contraindicated.

Thus, it was decided to perform space closure using a fixed orthodontic apparatus for subsequent placement of veneers on teeth 13 and 23 to substitute the maxillary central incisors and likewise on teeth 53 and 63 to substitute the maxillary lateral incisors. Teeth 14 and 24 were reshaped to assume the function of the maxillary canines and occlusal adjustments made to achieve lateral group function.

Despite the grinding of teeth for placement of veneers and full crowns, this treatment can be regarded as a conservative option, because teeth 53 and 63 were maintained thereby keeping the height and thickness of the bone plate intact for future placement of implants at a later date (17). In addition, the treatment took place over a short period of no longer than 1 year 6 months because the positions where teeth 13 and 23 had erupted were exploited.

## Conclusions

This clinical case illustrates the success of interdisciplinary treatment, combining orthodontics and esthetic dentistry to resolve the problem caused by agenesis of the four maxillary incisors. Maintaining the deciduous canines allowed preservation of the bone plate, given that the patient´s age precluded the placement of implants. The permanent maxillary canines retained in the position of the central incisors simplified the orthodontic treatment which, associated with porcelain restorations, contributed to an excellent outcome for this extremely rare case.
